# Exercise intervention for the risk of falls in older adults: A protocol for systematic review and meta-analysis

**DOI:** 10.1097/MD.0000000000024548

**Published:** 2021-02-05

**Authors:** Qin Zhang, Yi Liu, Dongze Li, Yu Jia, Wei Zhang, Bowen Chen, Zhi Wan

**Affiliations:** a Department of Emergency Medicine, Laboratory of Emergency Medicine, West China Hospital, and Disaster Medical Center; b School of Nursing, West China Hospital, Sichuan University, Chengdu, Sichuan, China.

**Keywords:** exercise, falls, meta-analysis, systematic review

## Abstract

**Background:**

Falls can easily lead to serious injury and even death in the older adults. Many exercise interventions, such as balance, flexibility, and endurance training have been shown to reduce the incidence of falls in this population. However, which mode of exercise is most beneficial for them remains unanswered.

**Methods:**

We will search the following databases as data sources: PUBMED, EMBASE, Cochrane Library, Wanfang, China knowledge Network (CNKI), Clinical Trials Database, and Science Network. Data extraction will be performed by two independent reviewers, who will discuss and resolve any differences, with the consensus of a third author. The RCTs will be selected according to prespecified inclusion criteria. The main outcome is the occurrence of a fall, and the secondary outcomes are the adverse consequences of a fall and a fall risk assessment index. If the heterogeneity test shows slight or no statistical heterogeneity, a fixed effect model will be used for data synthesis; otherwise, a random effect model will be used. We will develop a unified data extraction table including a number of parameters. The Cochrane cooperative bias risk tool will be used to evaluate the methodological quality of the selected RCTs. RevMan Manager v5.3 software and STATA v16.0 software will be used for data analysis. If enough randomized controlled trials (more than 10) are identified and selected.

**Conclusion:**

This protocol will be applied to synthesize the existing evidence so as to identify the most effective exercise program to prevent falls in the elderly.

**INPLASY registration number:**

INPLASY2020110008.

## 1 Introduction

The Prevention of Falls Network Europe defines a fall as an event in which a patient falls on the ground or onto a plane with lower initial position due to sudden, involuntary, and unintentional posture changes.^[1]^ With increasing age, the physical fitness of the older adults gradually decreases, and such a decline is usually manifested by falls and fall-related injuries. Falls are the third leading cause of chronic disability,^[2]^ with extremely high morbidity and mortality.^[3,4]^ Fracture is one of the common adverse consequences of falls,^[5,6]^ and falls also increase social costs and the burden on the health care system.^[7,8]^ Even when they do not cause physical harm, falls may lead to a decline in self-confidence and self-image, limited activity, and social isolation, which can accelerate the decline of functional organs in the older adults.^[9]^ Nearly 33% of the people aged over 65 years fall each year, and 22%-45% of them are injured, including 10% experiencing serious injuries, such as fractures or head injuries.^[10,11]^ The adverse effects of falls may lead to long-term functional decline, making it more difficult to take care of these older people.

The causes of falls can be classified into internal and external.^[12]^ The internal causes can be divided into age-related physiological changes and pathological causes, whereas the external causes are environmental factors, such as obstacles. Impaired balance is the main internal cause of falls, and may be related to the loss of muscle strength caused by lack of exercise or malnutrition. Therefore, compared with other interventions, exercise may be crucial in preventing falls in older individuals.^[13]^ The relationship between exercise interventions and fall risk reduction in the older adults has been studied. The World Health Organization guidelines on physical activity and sedentary behavior recommend that all older adults should exercise regularly.^[14]^ Recent evidence showed that exercise can reduce the fall rate by 23% in the older adults, and significantly reduce the risk of fall injuries, including fractures, head injuries, soft tissue injuries, and all other injuries requiring hospitalization.^[15]^ Many exercise interventions, such as balance, flexibility, and endurance training have been shown to be effective in reducing falls in the older adults.^[16,17]^

However, most of these studies focused exclusively on a specific mode or type of exercise, and the question of which exercise mode is most suitable for the older adults has not been answered. Therefore, the purpose of this systematic review is to synthesize the existing evidence to evaluate the effectiveness and safety of exercise in preventing falls in the older adults, and to identify the most suitable exercise model for this purpose.

## 2 Methods and analysis

The protocol has been developed according to the recommendations of the Preferred Reporting Items for Systematic Reviews and Meta-Analyses Protocols^[18]^ and was registered in the International Platform of Registered Systematic Review and Meta-analysis Protocols (https://inplasy.com) where it can be accessed under ID INPLASY2020110008. This systematic review and meta-analysis does not require ethical approval because it does not include information about participants and does not invade their privacy.

### 2.1 Eligibility criteria

#### 2.1.1 Types of studies

Randomized controlled trials (RCTs) of exercise interventions for falls in the older adults published in Chinese or English will be included in our review. Studies that are not RCTs, but meet the criteria of RCTs will also be included.

#### 2.1.2 Types of participants

Participants aged over 65 years will be included. However, older adults who are unable to exercise or have specific neurodegenerative diseases (such as severe visual impairment), dementia, stroke, or are undergoing palliative care will be excluded.

#### 2.1.3 Types of interventions

Our systematic review and meta-analysis will be based on the application of one or more exercise interventions in the experimental group, including single forms of strength exercise (general physical exercise, balance, gait, coordination) and 3-dimensional forms of exercise (Tai chi, dance and yoga). Exercise can be either a single intervention, or a part of multiple or multi-factor interventions. RCTs must include a control group treated with non-exercise interventions, such as imparting knowledge through lectures on routine fall-related health education, including how to prevent falls and related harm, the safe use of drugs, and mental health education for the older adults.

#### 2.1.4 Primary outcome

The main outcome in this study is the frequency of falls.

#### 2.1.5 Secondary outcomes

The secondary outcomes are adverse consequences of falls (such as fracture and death) and the fall risk assessment index.

### 2.2 Search methods

We will use the following databases: PUBMED, EMBASE, Cochrane Library, Wanfang, China National Knowledge Infrastructure, Clinical Trials Database, and Science Network as data sources. The following search keywords will be used:

1#: “fall” or “falls” or “falling”
2#: “older people” or “aged” or “elder” or “elderly”
3#: “physical exercise” or “exercise” or “training” or “exercise training” or “walking” or “Tai chi” or “dancing” or “yoga” or “qigong”

These will be combined as “1# and 2# and 3#.”

Two researchers (LY and ZQ) will manually and independently search for relevant research published from the date of establishment of each database. In order to avoid the omission of relevant literature, we will further search unpublished clinical trials and grey literature. Possible disagreements will be discussed and decided by consensus, with the intervention of the third researcher (JY) if necessary.

### 2.3 Data collection and analysis

#### 2.3.1 Selection of studies

**Figure 1:**
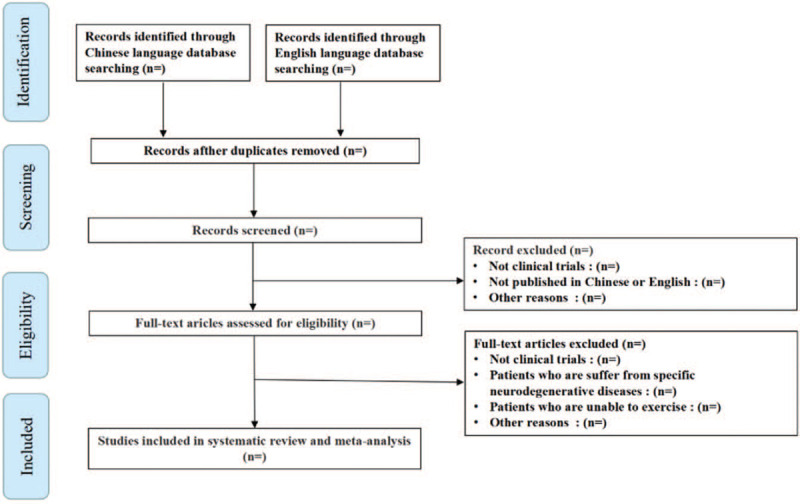
Flow diagram of study selection.

Two researchers (LY and ZQ) will independently select topics and abstracts to determine the literature that meets the criteria, and then read the full text to further screen the items and record the reasons for exclusions. Possible disagreements will be discussed and decided by consensus, with the intervention of the third researcher (JY) if necessary. The details of the study selection and identification process will be presented in a flow chart (Fig. 1).

#### 2.3.2 Data extraction and management

We will develop a unified data extraction table. The main fields will include author, year of publication, sample size, inclusion criteria, patient physical condition (health or disease), patient characteristics (age, sex, height, body mass index, history of disease (hypertension, diabetes, etc)), fall-related events (fall times, frequency), types of exercise, exercise program (aerobic exercise, aerobic exercise combined with anaerobic exercise), control group (non-exercise or other methods), follow-up time, and adverse events (death, fracture, hospitalization). The process will be completed by 2 researchers (ZQ and LY) independently. If the main data cannot be obtained from the original document, the communication author will be contacted by e-mail to obtain the original data, and failing this, the study will be excluded. All results will be reviewed by a third researcher (JY). Possible disagreements will be discussed and decided by consensus, with the intervention of the third researcher (JY) if necessary.

#### 2.3.3 Study quality assessment

The Cochrane cooperative bias risk tool^[19]^ will be used to evaluate the methodological quality of the selected RCTs. The literature will be divided into 3 quality grades: grade A (fully meet the standards, low possibility of bias), grade B (partially meets the standard, moderate possibility of bias), and grade C (does not meet the standards, high possibility of bias).

#### 2.3.4 Outcome measures

RevMan Manager v5.3 (The Cochrane Collaboration, Oxford, UK) software and STATA v16.0 software (Stata Corporation, College Station, TX) will be used for data analysis. Descriptive statistics will be obtained for demographic characteristics, with continuous data expressed as mean ± standard deviation, and categorical variables as frequencies. The effectiveness of the exercise intervention on falls in the older adults will be considered as categorical, and multivariate adjusted relative risks with 95% confidence intervals will be used to estimate the effect size. All hypothesis tests will be 2-sided, and *P*-values < .05 will be considered statistically significant.

#### 2.3.5 Assessment of heterogeneity

The heterogeneity among the studies will be judged by the combination of the χ^2^ test and I^2^ statistics. *P*>.05 and I^2^<50% will be taken to indicate statistical homogeneity among the studies, and a fixed effect model will be selected for meta-analysis in this case. If *P* ≤ .05 or I^2^ ≥ 50% will be considered to indicate statistical heterogeneity among the studies, and the random effect model will be selected for meta-analysis. Sensitivity analysis will be carried out at the same time. Descriptive analysis will be carried out when heterogeneity cannot be evaluated or when the effects cannot be combined.

#### 2.3.6 Assessment of reporting bias

If enough RCTs (more than 10) are identified and selected, funnel plots and the Egger or Begg tests will be used to assess potential publication biases. If there is no bias in the included studies, the points on the funnel chart are distributed symmetrically around the estimated true values of each independent study effect point, showing an inverted symmetrical funnel shape. If there is an offset, there is an asymmetric funnel diagram, and the more obvious the asymmetry, the greater the degree of bias, which may lead to overestimating the therapeutic effect.

#### 2.3.7 Subgroup analysis

If the number of studies included is sufficient, fracture risk subgroup analysis will be conducted according to mean age (young, middle, and older age groups), gender (female and male), body mass index (obese, overweight, normal, and underweight), intervention (single vs 3-dimensional forms of exercise), exercise time (more than 300 minutes per week, 150–300 minutes per week, less than 150 minutes per week), and exercise frequency (less than 3 days per week vs 3 or more days per week). The bias between the subgroups will be analyzed by interactive *P* test.

#### 2.3.8 Sensitivity analysis

Sensitivity analysis will be conducted using 2 methods to test the stability of meta-analysis results. The first consists in excluding each individual RCT, and the other is to change the Mantel–Haenszel fixed effect of the composite model to a random effect, or to change the random effect to a Mantel–Haenszel fixed effect.

#### 2.3.9 Ethics and dissemination

This protocol refers to a systematic review and meta-analysis; therefore, ethical approval is not required, as participants are not recruited and data are not collected from participants, and the study will not infringe the privacy or the rights of any individuals. The findings will be disseminated through peer-reviewed publications and academic gatherings.

## 3 Discussion

The mechanism of falls is related to the internal degenerative changes in the body. Exercise can improve muscle strength and balancing ability of the lower limbs in the older adults to prevent them from falling. Experts recommend strength, balance, and gait training under the guidance of medical professional, as it was shown that progressive muscle strengthening, balance training, and gait planning programs can effectively reduce the incidence of falls.^[20]^ Some systematic reviews and meta-analyses have described the role of exercise interventions in the older adults.^[21–23]^

However, previous studies have not indicated which exercise mode is the most effective in preventing falls in the older adults. This protocol puts forward a method of systematic review and meta-analysis to evaluate the effectiveness and safety of physical exercise in preventing falls in the older adults. The systematic review and meta-analysis will be performed and reported according to the Preferred Reporting Items for Systematic Reviews and Meta-Analyses guidelines, providing a high level of evidence on whether exercise significantly reduces the frequency of falls in the elderly. However, its limitations are those intrinsic to this type of analysis, since the credibility systematic reviews and meta-analyses is strongly affected by the quality of the included studies. Moreover, lack of data is also a common limitation of this kind of research, which may affect the quality of the results.

## Author contributions

**Conceptualization:** Dongze Li, Yu Jia.

**Funding acquisition:** Zhi Wan.

**Methodology:** Yu Jia, Wei Zhang.

**Resources:** Bowen Chen.

**Supervision:** Dongze Li.

**Writing – original draft:** Yi Liu.

**Writing – review & editing:** Qin Zhang.
